# The Institutional Treatment of Consumption

**Published:** 1916-05-20

**Authors:** 


### THE INSTITUTIONAL TREATMENT OF CONSUMPTION.

### II.—Routine Time-Tables and Diet-Scales.*

Sanatorium treatment aims at providing an abundance of fresh air, slight super-alimentation, and specific treatment in the shape of carefully controlled periods of rest and exercise, with or without the exhibition of tuberculin or other serums. Opportunity for taking advantage of all the available sunshine, both by those patients confined to bed as well as by those able to walk about, is an important item. Most patients soon realise that sanatorium treatment is very far removed from being a spell of luxurious loafing. They have to play a part more active than passive, and to submit to a discipline and a routine probably more strict than they imagined. But it is practically impossible to imitate sanatorium treatment in any other place, even with the best intentions in the world. In a sanatorium the patient has the stimulus of seeing those in whom the disease has become arrested and who are engaged in active work. Then he will not mind using the pocket sputum flask, which at first is always somewhat disagreeable, when around him are others doing likewise. But the chief point is obviously the great and continuous medical supervision which can be and is exercised in an institution. A rise of temperature can be promptly treated by "immobilising" the patient.

The routine of a day in a sanatorium varies a little in different places, but the following time-table will serve to give an average idea:

| | |
|---|---|
| A.M. | |
| 7 or 7.30. | Temperature and pulse taken. Washing or bath, and dressing. |
| 8 or 8.30. | Breakfast. |
| 9 to 9.30. | Rest or recreation. |
| 9.30 to 12. | Rest or exercise, or work as prescribed. Doctor's visit. |
| P.M. | |
| 12 to 1. | Rest for all patients; no talking or smoking. Temperature taken. |
| 1 to 2 | Dinner hour. |
| 2 to 2.30. | Rest or recreation; smoking allowed. |
| 2.30 to 4.30. | Rest, exercise, or labour as prescribed. |
| 4.30 | Tea. |
| 5 to 6. | Recreation. |
| 6 to 7. | Rest lying down; temperature and pulse taken. |
| 7 to 7.30. | Supper. |
| 7.30 to 8.30. | Recreation. |
| 8.30. | Bed. |
| 9. | Lights out. |

It is usual to keep in bed all new patients for at least four days, and often for a week, in order to see if the temperature remains steady, or what is its range while the patient is resting. The physician is thereby given an adequate opportunity of examining the chest and of forming an opinion on the type of case. Temperature records are of first importance in sanatoria work. Rectal temperatures are more accurate, and some authorities insist that these are the only ones of use in treating patients on strict sanatorium lines, but there are a great many institutions in this country where mouth or axilla temperatures are the established routine. When high temperatures are being recorded the difference between those taken in the mouth and those in the rectum is not great, but round about the normal the differences are usually greater, the rectal temperatures tending to be higher. Into questions of clinical thermometry it is not proposed here to enter, and it must suffice to mention that mouth temperatures cannot be reliable if taken out-of-doors in cold weather or by an open window, and that axillary temperatures are apt to be misleading when the patient is thin or has a moist axilla. Ten minutes or even a quarter of an hour should be allowed for mouth records with ordinary hospital thermometers. Before breakfast the temperature should not be above normal if the patient has had a restful night. After exercise the temperature is naturally raised, but the criterion of febrility is an unduly prolonged delay in falling to normal after a period of rest.

A patient having been classed as febrile is an indication for prescribing rest, a term which may denote anything from rest on a couch to complete and absolute "typhoid" rest when the patient is not allowed to do anything for himself. The fever is an expression of the activity of the tubercular processes, and is a measure of the absorption of toxins; these also affect the appetite, and a good appetite is a very important thing. Hence it is sought to diminish by rest the washing into the blood-stream of these toxins, and incidentally to spare the heart (which is working at an increased rate), to lessen cough, which strains the damaged lung, and to reduce the liability to nausea or vomiting.

One of the most obvious features of pulmonary tuberculosis is the wasting which is the consequence of the anorexia and constitutional disturbances. To this feature we owe the name "consumption." Hence diet was assigned a place of paramount importance from the earliest days of sanatorium treatment, and actual stuffing of the patients became the recognised thing. Extra milk to the extent of three or four pints a day, in addition to four liberal meals, was forced upon the patients, who became so fat as to suffer from dyspnœa, and decidedly lazy as well. Nowadays it is recognised that it is superfluous and actually detrimental to give milk before breakfast and between meals; the stomach never gets a prolonged rest, and the appetite for the proper meals is continually vitiated. Moreover, some regard must be paid to the conditions to which the patient will have to return when at home. Three meals a day are sufficient, but it is customary to allow an afternoon tea in almost all sanatoria. As we are considering patients in sanatoria maintained wholly or in part by public funds, it is easy to give a more or less typical dietary. In "full price" sanatoria a late dinner is often provided, and in others the supper varies from a moderately substantial meal to a glass of milk with a biscuit. The following is

* Article I. of this series appeared in The Hospital of May 6, 1916.

the diet allowed at a sanatorium controlled by a county council:—

BREAKFAST.—Porridge, $\frac{1}{2}$ pint (2 oz. oatmeal); sugar, $1\frac{1}{2}$ oz.; bacon, 2 oz. (uncooked); or one egg, with $1\frac{1}{2}$ oz. ham or bacon; or one herring or bloater or kipper or piece of haddock; bread, 4 oz.; butter, $\frac{1}{2}$ oz.; tea; milk, $\frac{1}{4}$ pint, with porridge and tea.

DINNER.—Meat, 12 oz. (as purchased), rabbit and pork; or fish, 12 oz. (as purchased), preferably fried in fat; potatoes, 8 oz.; vegetables, greens with roast meat twice a week, beans, lentils, carrots, etc., with boiled meat; meat may be given in pies and puddings; sweets—milk puddings, suet puddings, or tarts.

TEA.—Bread, 8 oz.; butter, $\frac{1}{2}$ oz.; jam, 2 oz.; or 6 oz. bread, with cakes, but no jam.

SUPPER.—Half-pint of milk; bread, 3 oz.; butter, $\frac{1}{2}$ oz., with milk pudding and $1\frac{1}{2}$ oz. cheese; or $\frac{1}{2}$ pint vegetable soup and cheese; or sausage, liver, tinned salmon, or tripe (3 oz. each), or potted meat.

This is a fairly liberal dietary, and there are not a few sanatoria at which, for the duration of the war, at any rate, supper "extras" have been cut down to cheese or porridge. At a large institution near London no eggs are provided in the standard diet for the winter months, and at one breakfast a week two bananas constitute the "extra." Several sanatoria allow for a half-pint of milk for every patient before breakfast, and some give another glassful at 10 A.M. If, as is sometimes the case, breakfast is not partaken of until nearly two hours after the milk is drunk on awakening, this early ration of milk is permissible, but for patients on a full or "meat" diet extra milk should not be encouraged. "Fish" or "fowl" diets may be substituted in special cases, while "light" diet and "milk" diet are naturally almost always for hospital cases and need no description.

